# Scoping review on diagnostic criteria and investigative approach in sepsis of unknown origin in critically ill patients

**DOI:** 10.1186/s40560-022-00633-4

**Published:** 2022-09-11

**Authors:** Lowell Ling, Oliver Oi Yat Mui, Kevin B. Laupland, Jean-Yves Lefrant, Jason A. Roberts, Pragasan Dean Gopalan, Jeffrey Lipman, Gavin M. Joynt, for the CaBoOM investigators, Tom Stelfox, Daniel Niven, Rameiya Paramalingam, Derek Vonderhaar, Ross Freebairn, Gavin M. Joynt, Lowell Ling, Patricia Leung, Dean Gopalan, Jean Yves Lefrant, Sophie Lloret, Loubna Elotmani, Jason A. Roberts, Jeffrey Lipman, Kevin B. Laupland, Cheryl Fourie, Renee Saba, Dougal Carlisle, Felicity Edwards

**Affiliations:** 1 Department of Anaesthesia and Intensive Care, The Chinese University of Hong Kong, Hong Kong, SAR China; 2 Faculty of Medicine, The Chinese University of Hong Kong, Hong Kong, SAR China; 3 Department of Intensive Care Services, Royal Brisbane and Women’s Hospital, Brisbane, QLD Australia; 4 Queensland University of Technology (QUT), Brisbane, QLD Australia; 5 UR-UM103 IMAGINE, University of Montpellier, Division of Anesthesia Critical Care, Pain and Emergency Medicine, Nîmes University Hospital, Montpellier, France; 6 University of Queensland Centre for Clinical Research (UQCCR), Faculty of Medicine, The University of Queensland, Brisbane, Australia; 7 Department of Intensive Care Medicine and Pharmacy, Royal Brisbane and Women’s Hospital, Brisbane, Australia; 8 Discipline of Anaesthesiology & Critical Care, Nelson R Mandela School of Medicine, University of KwaZulu Natal, Durban, South Africa; 9 Jamieson Trauma Institute, Royal Brisbane and Women’s Hospital, Brisbane, QLD Australia

**Keywords:** Sepsis, Search, Investigation, Examination, History, Imaging, Infection, ICU, Diagnosis, Criteria

## Abstract

**Background:**

Up to 11% of critically ill patients with sepsis have an unknown source, where the pathogen and site of infection are unclear. The aim of this scoping review is to document currently reported diagnostic criteria of sepsis of unknown origin (SUO) and identify the types and breadth of existing evidence supporting diagnostic processes to identify the infection source in critically ill patients with suspected SUO.

**Methods:**

A literature search of Embase, MEDLINE and PubMed for published studies from 1910 to August 19, 2021 addressing the topic of SUO was performed. Study type, country of origin according to World Bank classification, diagnostic criteria of sepsis of unknown origin, and investigative approaches were extracted from the studies.

**Results:**

From an initial 722 studies, 89 unique publications fulfilled the inclusion and exclusion criteria and were included for full text review. The most common publication type was case report/series 45/89 (51%). Only 10/89 (11%) of studies provided a diagnostic criteria of SUO, but a universally accepted diagnostic criterion was not identified. The included studies discussed 30/89 (34%) history, 23/89 (26%) examination, 57/89 (64%) imaging, microbiology 39/89 (44%), and special tests 32/89 (36%) as part of the diagnostic processes in patients with SUO.

**Conclusions:**

Universally accepted diagnostic criteria for SUO was not found. Prospective studies on investigative processes in critically ill patients managed as SUO across different healthcare settings are needed to understand the epidemiology and inform the diagnostic criteria required to diagnose SUO.

**Supplementary Information:**

The online version contains supplementary material available at 10.1186/s40560-022-00633-4.

## Background

Sepsis is life threatening organ dysfunction due to abnormal host response to an infection [1]. Treatment of underlying infection with appropriate early antimicrobials and source control are cornerstones of sepsis management [2]. The use of antimicrobial agents is guided by identification of causative organisms whilst timely source control relies on locating a surgical source of infection. However, culture negative sepsis is found in up to one half of septic patients [3–8]. Lack of confirmatory microbiology may be caused by the use of antimicrobials prior to cultures, inadequate sampling or fastidious organisms [9–11]. Furthermore, it has been reported that 2–11% of critically ill patients have sepsis of unknown origin (SUO) [4, 12–17]. In these patients, not only are microbiological investigations negative, the site of infection is also unclear. Failure to identify the source of sepsis, or identify the offending microorganism is associated with higher risk of severe organ dysfunction and mortality [8, 18]. To complicate matters further, patients may instead have non-infective cause of organ dysfunction rather than sepsis. The key question to determine in patients with suspected SUO is whether an infection is the cause of the manifested systemic inflammation and organ dysfunction.

For these reasons a systematic and comprehensive workup to identify the underlying source of infection or non-infective pathology is often recommended in patients with suspected SUO [19]. However, there is lack of empirical data to guide the optimal diagnostic approach to identify the underlying infection source in these patients. Observational data suggests that this is a common problem as only 74% of patients with suspected septic shock have an infection source identified within 24 h of ICU admission [16]. Additional, albeit un-protocolized workup, will result in a further 7% who will have the source of infection identified after the initial 24 h of admission for shock. Nevertheless, this study showed that further diagnostic workup over time will lead to a definitive diagnosis in a significant portion of patients initially thought to have SUO [16]. Yet, if identification of infective cause of sepsis is dependent on time and extent of investigation, then when should patients be considered to have SUO?

The primary objective of this scoping review is to document currently reported diagnostic criteria for SUO. The secondary objective was to characterize the types and breadth of existing evidence supporting diagnostic processes to identify the cause of infection in critically ill patients with suspected SUO.

## Methods

### Study design and search strategy

This scoping review was designed using the PRISMA Extension for Scoping Reviews and the research protocol is published online (10.6084/m9.figshare.20493444.v1) [20]. The key elements of the research question were as follows: the “population” was adult patients with SUO, “concept” was diagnostic criteria and investigative approach and “context” was intensive care unit. We performed a literature search of Embase, MEDLINE and PubMed for published studies since 1910 on August 19, 2021 using the search strategy shown in Additional file 1. Studies published prior to the first consensus definition of sepsis in 1992 was included in this review. Our rationale was that the need of a diagnostic approach to identify a suspected infective cause in patients presenting with severe inflammation or organ dysfunction was recognized much earlier [21]. Furthermore, although the definition of sepsis has changed over the years, the association between infection and life-threatening organ dysfunction and inflammation has remained consistent. Thus, this scoping review aimed to document the diagnostic criteria used to define “suspected infection” in patients with SUO [1]. An iterative search strategy was utilized, and the final search strategy was based on content extracted from included studies. The term “adult” was not used in the search strategy as it resulted in very limited studies for review. We included all studies published in English, Chinese and German.

### Eligibility criteria and selection process

Studies were included if they included: (1) diagnostic criteria for SUO or (2) described quantitative or qualitative diagnostic methods to establish an infective cause of sepsis which was initially unknown. Studies must include critically ill patients managed in the ICU. Articles that were limited to describing patients with primary bacteraemia or fungaemia of unknown source were excluded. This exclusion was based on the rationale that these patients had a confirmed infection, despite an unclear physical origin of the pathogen detected in blood cultures. This group of patients is fundamentally different to the group of patients who have suspected SUO, where both the infection site and pathogen are unknown, or who may not have any infection. It is evident that the latter group require a substantially different diagnostic approach, which is the focus of this scoping review. Studies focused on pyrexia of unknown cause in non-critically ill patients were also not included. Although the methods of investigation may overlap to some degree, our rationale was that critically ill ICU patients with SUO represent a different population, with different urgency relating to decision making. Compared to stable patients with pyrexia of unknown origin, diagnosis of the presence of infection in ICU patients with suspected SUO presents a constrained opportunity for lengthy and first-person history, often demonstrates clouded clinical signs and a restricted time-frame for implementing progressively more invasive, often serial, and time-consuming investigations to make a diagnosis and initiate appropriate treatment.

Two independent researchers (LL and OOYM) used the eligibility criteria described and screened the title and abstracts of studies gathered from the search. Full texts of initially selected studies were reviewed to confirm eligibility. Disagreements were resolved by discussion, and if remained unresolved an adjudicator made the final decision.

### Data extraction

A data extraction chart was developed by two reviewers (LL and OOYM) to extract information from included studies. This chart was constructed in the following steps. First, a template to extract data on characteristics of the article including author, publication year, country/territory of origin, language, title, and type of study was constructed. Country/territory of origin was defined as the location of the study population or address of the corresponding author. Second, a free text entry box was created to document the following: the presence of diagnostic criteria for SUO, clinical findings, extent of investigations, and the timing and duration of diagnostic method required prior to diagnosis of SUO. Diagnostic criteria for SUO was recorded when there was direct reference to a stated set of criteria for individual classification, or study inclusion criteria for patients. Third, all studies were reviewed for diagnostic thematic processes used to identify the infection source or an alternative non-infective pathology. We observed that the main processes described included history, examination, imaging, microbiological sampling and special tests. These themes were then added to the data extraction template and the corresponding data extracted. Critical appraisal of potential bias and heterogeneity was not performed.

### Statistics

Descriptive statistics using percentages were used to summarize extracted information. Studies were grouped by World Bank income classification to show the distribution of results by different resource settings. Inter-rater reliability between reviewers on inclusion and exclusion of studies was assessed using Kappa statistic [22].

## Results

**Fig. 1:**
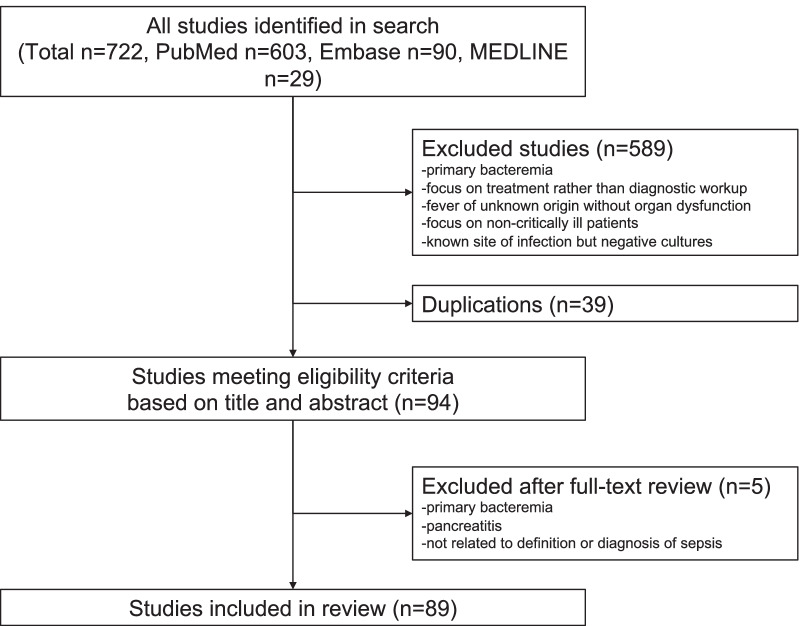
Flow chart of study inclusions

We found a total of 722 potentially relevant studies, with 603 from PubMed, 90 from Embase and 29 from MEDLINE (Fig. 1). After screening the titles and abstract, 133 met the inclusion and exclusion criteria. Subsequently, 39 duplicated studies were excluded, and therefore, 94 studies were included for full-text procurement. Of these 94 studies, only 63 studies had full accessible text, with the remaining 31 studies reviewed in abstract or conference proceeding format. After full text review, 5 studies not related to the inclusion criteria were excluded, because they were not related to diagnostic criteria or diagnosis of SUO, focused on diagnostic workup on patients with primary bacteraemia and described case of pancreatitis rather than sepsis. Therefore, a final collection of 89 studies was included in the scoping review (Additional file 2). The inter-rater agreement for inclusion and exclusion of studies prior to discussion was good between the two reviewers (*κ* = 0.83, 682/722 agreement). After discussion, the two reviewers agreed on all study inclusions and a third reviewer was not required for adjudication.

### Characteristics of studies

An overview of the included studies is shown in Table 1. All included papers were published from 1982 to 2021, with 33/89 (37%) published between 2015 and 2019. The most common publication type was case reports or case series 45/89 (51%). Included studies originated from 23 different countries/territories, with the United States contributing to the greatest number of studies at 28/89 (31%) (Table 2). The majority of the studies 82/89 (92%) were from high income countries/territories, and none were from low-income countries/territories. Studies included in the final review were either in English 85/89 (96%) or German 4/89 (4%).

**Table 1:** General characteristics of included studies

	*n* = 89 (%)
Publication year	
Before 2005	19 (21)
2005–2009	7 (8)
2010–2014	22 (25)
2015–2019	33 (37)
After 2019	8 (9)
Publication type	
Case reports/series	45 (51)
Review	12 (13)
Prospective cohort	19 (21)
Retrospective cohort	11 (12)
Randomized controlled trial	1 (1)
Systematic review	1 (1)

**Table 2:** Distribution of studies by income group

Income group	Country/Territory	Number of studies
High income	Australia	2
	Austria	1
	Belgium	2
	Canada	1
	Czech Republic	1
	France	8
	Germany	15
	Greece	2
	Italy	5
	Japan	1
	Korea	1
	Netherlands	3
	Poland	1
	Portugal	1
	Spain	2
	Taiwan	1
	United Kingdom	7
	United States	28
Upper middle income	South Africa	1
	Thailand	1
	Turkey	1
Lower middle income	India	3
	Nepal	1
Low income	–	–

Country/territory was based on the study population or address of the corresponding author

### Diagnostic Criteria of SUO

Criteria used to define SUO were not provided in 79/89 (89%) of included studies. Even in the remaining 10/89 (11%), there was no consensus on the diagnostic criteria of SUO, or reference to known consensus diagnostic criteria (Table 3) [23]. The criteria, when present, often described as failure to identify source of sepsis despite “radiological—including CT—and microbiological technology, and systematic diagnostic workups” or after “extensive diagnostics” [24, 25]. However, the breadth and depth of examination and investigations that constitute a comprehensive workup was rarely described explicitly. Most diagnostic criteria focused on laboratory and radiological investigations, whilst the extent and thoroughness of medical history was never mentioned as a requirement. Clinical signs or examination was stated as necessary in 6/89 (7%) of publications but none explained the scope of negative findings required to fulfill the diagnostic criteria for SUO [26–31]. Even for microbiological investigations, the requirement for all of routine blood, urine and respiratory bacterial cultures was mentioned in only 3/89 (3%) of studies [28, 32, 33]. In addition, 3/89 (3%) of studies classified patients as SUO if investigative work up was negative after a cutoff time of 24 to 48 h [16, 28]. None of the diagnostic criteria mentioned whether non-infective differential diagnoses, if any, should be investigated for before attributing a patient’s clinical condition to SUO.

**Table 3:** Published diagnostic criteria of SUO

Agarwal et al*.* 2006 [23]	“…suspected sepsis with no apparent infection at any site and negative blood cultures, along with the intensive care physician’s decision to start empiric antibiotics”
Contou et al*.* 2016 [16]	“septic shock and with no clear diagnosis (lack of both a source of infection and microbiological documentation) within the first 24 h of vasopressor introduction”
Fort et al*.* 2018 [25]	"radiological—including CT—and microbiological technology, and systematic diagnostic workups, the source of the sepsis is not always definitely identified"
Hulst et al*.* 2019 [88]	“clinical examination, extensive microbiological and diagnostic testing, such as computed tomography (CT), the septic focus cannot always be detected”
Kelly et al*.* 2000 [27]	"in patients with no physical or laboratory evidence of a source"
Kluge et al*.* 2012 [32]	"standardized diagnostic workup including microbiological evaluation (cultures of blood, urine, and respiratory secretions), chest X-rays, CT scanning, and transesophageal echocardiography according to the standard departmental protocol…when clinical signs and/or laboratory and/or imaging findings to identify the source of infection were inconclusive"
Lee et al*.* 1991 [33]	“complete clinical, imaging, and laboratory tests had ruled out other septic sources. Tests performed in all patients to exclude other sources of sepsis included multiple blood, urine, and sputum cultures; cultures of tips of central line catheters; abdominal CT scans; and serial chest radiographs”
Mandry et al*.* 2014 [28]	“after 48 h of extensive investigations. A unique procedure was not imposed for these diagnostic investigations, as they were dependent on clinical context. However, in addition to clinical examination, chest X-ray and conventional laboratory investigations (blood cultures, urine analysis, detection of soluble antigens, bronchoalveolar lavage fluid [BALF] culture, serology), most patients benefited from an echocardiography (transthoracic and/or transesophageal), an abdominal echography and whole body CT-scan before inclusion”
Minoja et al*.* 1996 [29]	“extensive diagnostic workup to localize infection, including a careful analysis of clinical and intraoperative findings, microbiological and serological data, X-rays, and US and CT images. Patients with suspected pneumonia underwent fiberoptic bronchoscopy, with bronchoalveolar lavage and protected specimen brush” and “radiologically identified deep-seated fluid collection, suspected of being but not demonstrated to be infected”
Velmahos et al*.* 1999 [31]	“no other test confirmed an infectious focus that could explain the clinical picture or if signs were not adequately explained by the existing evidence (persistent sepsis despite culture-specific antibiotics with adequate blood levels of known respiratory tract infection)”

### History

Despite the universal absence of history as a component of the diagnostic criteria for SUO (when provided), 30/89 (34%) of studies discussed the merits of specific, focused history taking. Most of these studies describe how a particular aspect of history helped direct further investigations to confirm the infective source or to suggest alternative diagnoses [34–40]. Important aspects of history taking included travel history which may reveal exposure to endemic pathogens, such as malaria and rickettsia [34, 37]. Other studies highlighted the importance of drug history which may reveal use of drugs which may cause symptoms and signs that mimic sepsis [35, 36, 38]. Non-specific symptoms of prolonged fever, myalgia and weight loss were described as suggesting malignancy, rheumatological disease or hematological disorders [41–46]. None of the studies described what a thorough and comprehensive history should encompass to best investigate SUO.

### Examination

Although 23/89 (26%) of studies described specific signs on examination that provided clues to infective source, none of them outlined what would be considered a systematic examination. Isolated and specific recommendations were, however, made. Examination of the oral cavity and soft tissues in the neck may suggest localized infection or even Lemierre’s syndrome [40, 47]. Furthermore, examination based on context such as finding soft issue infection in patients with burns may be helpful [47, 48]. Repeated examinations were helpful to identify source of infection in patients who did not initially have localizing signs [49]. Nonspecific signs such as lymphadenopathy or maculopapular rash may suggest lymphoma or rheumatological conditions, such as Adult Onset Still’s Disease, respectively [50, 51]. Seven studies noted the significance of hepato- and/or splenomegaly which suggests underlying hematological disorders [41, 44–46, 52–54]. None of the studies reported inter-observer variability in identification of clinical signs.

### Imaging

Utility of imaging was discussed in 57/89 (64%) of studies, specifically to identify the source of sepsis. Point of care ultrasound was demonstrated to be useful to assess many body compartments including pulmonary, urinary, biliary and musculoskeletal [55–57]. Computed tomography (CT) was often reported as a helpful screening tool to increase diagnostic yield when source of sepsis is unknown [26, 31, 39, 49, 51, 57–63]. Along with plain radiographs, ultrasound and echocardiography, studies have generally recommended that it is essential to perform a screening CT scan before a diagnosis of SUO is made [25, 28–30, 32, 33]. Furthermore, the addition of positron emission tomography–CT (PET–CT) has been shown to be a key investigation resulting in a positive diagnosis in critically ill patients with suspected SUO [25, 28, 32, 64–66]. One study showed that 99 m Tc labeled white cell has high sensitivity (95%) and specificity (91%) for identifying site of infection in trauma and surgical ICU patients with SUO [29].

### Microbiological Culture Sampling

Microbiological cultures were described in 39/89 (44%) of studies. Blood culture was considered mandatory in only 6 studies [23, 28, 32, 33, 66, 67]. Overall, none of the studies specified the criteria for properly performed cultures but one study mentioned that a minimum of two peripheral blood cultures was needed [23]. Beyond cultures from blood, respiratory and urinary tract, cultures from catheter tips and hubs, sinus lavage and bronchoalveolar lavage were often described as part of the workup to establish SUO [16, 23, 28, 29, 32, 35, 37, 58, 60, 61, 64, 67–69]. Culture from bone marrow may also be helpful [70]. Lumbar puncture may be undertaken in patients with neurological symptoms [36, 71].

### Special Tests

Special investigations were described in 32/89 (36%) studies and included advanced microbiological tests, special techniques to obtain microbiological samples and other tests performed to look for alternative diagnoses. Streptococcus pneumoniae and legionella pneumophila urine antigens, thyroid hormones, cortisol and toxicology screening were also part of the diagnostic workup described by some authors [35, 36, 72]. Serology for rickettsial infections, screening for schistosomiasis, leishmaniasis were used in areas, where the pathogens are endemic [37, 42]. Interestingly, malaria screening may be warranted even when patients have not travelled to endemic areas as rare events may allow vector borne diseases to transmit in non-endemic areas [73]. Skin biopsy of rashes/lesions may help rule out septic emboli whilst histology may facilitate diagnosis of rheumatological or drug sensitivity disorders [35].

Monospot test and Epstein–Barr Virus polymerase chain reaction (PCR) may be useful as part of the diagnostic workup for hemophagocytic lymphohistiocytosis (HLH) [44, 62, 74]. QuantiFERON-TB Gold to screen for mycobacterium tuberculosis was mentioned in one study [74]. Five studies mentioned the need for human immunodeficiency virus testing [25, 37, 39, 42, 53]. Procalcitonin was tested in 10/89 (11%) studies as a marker suggestive of bacterial sepsis, although it cannot help to localize the site of infection [18, 30, 34, 38, 41, 52, 62, 65, 66, 75]. Bone marrow examination and biopsy of lesions or lymph nodes were used to rule out malignancy or HLH [41, 44–46, 50–53, 62, 74, 76]. Extremely high concentrations of ferritin may be helpful to screen for HLH [33, 41, 44–46, 52–54, 62]. Utilization of percutaneous cholecystostomy or diagnostic laparoscopy was found to be helpful in patients with SUO in certain instances when other clinical, laboratory and imaging tests were negative [30, 33, 77, 78]. A diagnostic autoimmune panel has been recommended to screen for alternative diagnoses, such as granulomatous polyangiitis or other rheumatological disorders [71].

## Discussion

To our knowledge this is the first comprehensive scoping review seeking to document the diagnostic criteria of SUO and characterize the existing evidence supporting diagnostic processes to identify the infection source in critically ill patients with suspected SUO. The systematic literature search identified 89 relevant studies of which the majority were case reports that almost exclusively originated from high income countries/regions. Universal diagnostic criteria for SUO were not found. There is also currently no standardized diagnostic approach for patients with suspected SUO, although common investigative processes included history, physical examination, imaging, microbiological investigations, and special tests.

Surprisingly, universally accepted diagnostic criteria for SUO were not found despite reported data suggesting 2–11% of septic patients have an unknown infectious source [4, 12–17]. We found that common elements described in different criteria for SUO included negative clinical, microbiological, imaging and special tests (Table 3). Yet, what constitutes a thorough clinical history and examination to localize the infective source was not standardized. Similarly, only 3% of studies listed sputum, blood and urine bacterial cultures as essential microbiological investigations, yet it would seem reasonable that they should be. Furthermore, the need for fungal and parasitic workup was not described or mentioned in any of the diagnostic criteria reviewed. The use of serological tests was mentioned, but the types of serology to be tested were not specified. Thus, using these current loose criteria may result in the premature conclusion that patients have SUO if non-bacterial microbiological tests were not performed, especially since 21% of infections in ICU are viral, parasitic or fungal in origin [79]. In addition, CT imaging was frequently listed as a criterion, but which body part(s) should be scanned is often not specified. Overall, the published diagnostic criteria of SUO included in this review (Table 3) are focused on the prerequisite infection workup in SUO without an explicit mention on what constitutes sepsis. This suggests most diagnostic criteria of SUO are concerned with how best to define “suspected infection” with the assumption that infection is responsible for the life threatening organ dysfunction and inflammation in sepsis.

Investigations such as microbiological culture require time for results to become available, and delay in diagnosis may be incurred for patients in smaller centers who require transfer for imaging, such as CT [80]. Indeed, an allowable time delay of 24 to 48 h was part of the criteria in 2 of the included studies [16, 28]. Thus, patients initially managed as suspected SUO may have a source identified or an alternative diagnosis established as time progresses [16]. The diagnosis in patients suspected to have SUO should be treated as an evolving, working diagnosis which is time dependent.

There is currently no consensus on the type, extent and sequence of investigative processes, or the minimal time interval required before a patient should be considered to have SUO. To better define this common clinical problem, empiric data from prospective studies are required to inform the drafting of evidenced-based diagnostic criteria for SUO in critically ill patients. The current information gap includes three key areas. First, the proportion of patients who on ICU admission have suspected sepsis but the infection site and pathogen are unknown. Second, the time interval from suspicion of sepsis to confirmation of infection. Third, the time sequence and breadth of diagnostic workup commonly taken to locate the infection or establish an alternative diagnosis. With these data it may be possible to construct a criteria to epidemiologically define patients with SUO as a diagnosis, likely by exclusion after protocolized workup has failed to identify a source of infection or establish a non-infective alternative diagnosis of life threatening organ dysfunction.

Development of a standard, protocolized diagnostic approach in patients with suspected SUO may reduce lapses in workup efficiency and minimize missed opportunities to identify the infection source or an alternative diagnosis. Our search found that core components of common investigative processes include: history, clinical examination, imaging, microbiological tests and special tests. Apart from localizing symptoms, one study highlighted the importance of social history, where a woman living in Frankfurt without travel history was suspected to have contracted malaria by living near the airport [73]. Although systematic examination is advocated, we were unable to find a set of examination components for this task. Over 50% of the included studies mentioned imaging tests in their diagnostic approach. Comparative studies have generally found CT to be superior to USG in detecting intra-abdominal sepsis, particularly in patients with recent surgery [81, 82]. Interestingly, at least 6 studies discussed the merits of PET–CT in identifying infection source in critically ill patients with SUO [25, 28, 32, 64–66]. Whilst microbiological culture is currently the gold standard to confirm infection, polymerase chain reaction and next generation sequencing pathogen detection may offer superior sensitivity for fastidious bacterial organisms, prior antimicrobial therapy or non-bacterial pathogens [83–85].

Finally, 92% of the available literature on diagnostic criteria of sepsis of unknown cause and investigative approach is from high income countries/regions alone with none from low-income countries. Advanced diagnostic tests and procedures may not always be available to all clinicians. Furthermore, variation in case-mix and endemic pathogens will likely determine the specific investigations required for different healthcare settings [86, 87]. An example would be the need to routinely screen for malaria in many parts of sub-Saharan Africa, whereas malaria screen would be of much lower priority in northern European countries unless there is a travel history. Therefore, it may be necessary to tailor the protocolized investigate workup in patients suspected of SUO based on the healthcare setting, country income, population structure, comorbidities, resource and local pathogens.

Our scoping review has a few important limitations. First, we restricted our search strategy to SUO in ICU patients which may have reduced the volume of available evidence. However, we decided not to include ward patients with mild organ dysfunction, because conceptually, they represent a different population with less clinical urgency and more time for investigations for suspected SUO. Second, because of limited relevant literature identified, we included case reports, abstracts and conference proceedings in this review. Review articles were also included to report expert opinion as there was lack of empirical data to guide the diagnostic approach in SUO. Although we were unable to systematically assess heterogeneity, the limited range of high level of published evidence showed that there is likely significant bias. Third, we used ICU admission to indicate the presence of critical illness and organ dysfunction as a study inclusion criteria, because a substantial number of studies did not provide sufficient information about organ dysfunction to allow a precise definition of sepsis or sepsis severity. Fourth, we were unable to separately summarize the diagnostic approach used for SUO patients presenting from the community or hospital setting, because the number of studies was limited.

## Conclusions

A universally accepted diagnostic criteria of SUO was not found. Prospective studies on investigative processes in critically ill patients managed as SUO across different healthcare settings are needed to understand the epidemiology and inform the diagnostic criteria of SUO.

## Supplementary Information

**Additional file 1. **Search Strategy**.** Search strategy used for Embase, PubMed and MEDLINE in this scoping review.

**Additional file 2. **Summary of Included Studies**.** Background information and extracted data from included studies.

**Additional file 3.** Diagnostic Workup Extraction**.** Extraction of data on diagnostic thematic processes from articles.

## Data Availability

All data generated or analyzed during this study are included in this published article [and its supplementary information files].

## Abbreviations

CT: Computed tomography
HLH: Hemophagocytic lymphohistiocytosis
ICU: Intensive care unit
USG: Ultrasonography
PCR: Polymerase chain reaction
PET–CT: Positron emission tomography–computed tomography
SUO: Sepsis of unknown origin
