# Supplementary material for: Scoping review on diagnostic criteria and investigative approach in sepsis of unknown origin in critically ill patients
Source: J Intensive Care. 2022 Sep 11;10:44. doi: 10.1186/s40560-022-00633-4 (PMC9465866; doi:10.1186/s40560-022-00633-4)
Supplement: Supplementary file 1 — Additional file 1. Search Strategy. Search strategy used for Embase, PubMed and MEDLINE in this scoping review. [file 40560_2022_633_MOESM1_ESM.docx]

**Search Strategy**

**Database: Embase 1910 to August 19, 2021**

Search Strategy:

--------------------------------------------------------------------------------

1 Sepsis/ or sepsis.mp. (232534)

2 unknown source.mp. (833)

3 unknown source.m_titl. (87)

4 fever of unknown origin.mp. or exp "Fever of Unknown Origin"/ (8034)

5 sepsis of unknown origin.mp. (142)

6 sepsis of unknown origin.m_titl. (10)

7 sepsis of unknown cause.mp. (19)

8 sepsis of unknown cause.m_titl. (3)

9 sepsis of unknown source.mp. (15)

10 sepsis of unknown source.m_titl. (0)

11 5 or 6 or 7 or 8 or 9 or 10 (174)

12 2 or 3 or 4 (8858)

13 1 and 12 (707)

14 11 or 13 (862)

15 Diagnosis/ or diagnosis.mp. (5688353)

16 investigation.mp. (779374)

17 test.mp. (3278265)

18 examination.mp. or Physical Examination/ (1435095)

19 History/ or history.mp. (1447607)

20 approach.mp. (1943104)

21 imaging.mp. (2011119)

22 15 or 16 or 17 or 18 or 19 or 20 or 21 (12159765)

23 14 and 22 (589)

24 critically ill.mp. or Critical Illness/ (102749)

25 Intensive Care Units/ or ICU.mp. or Critical Care/ (293679)

26 24 or 25 (348262)

27 23 and 26 (90)

***************************

**Database: Ovid MEDLINE(R) 1946 to August 19, 2021**

Search Strategy:

--------------------------------------------------------------------------------

1 Sepsis/ or sepsis.mp. (119870)

2 unknown source.mp. (442)

3 unknown source.m_titl. (40)

4 fever of unknown origin.mp. or exp "Fever of Unknown Origin"/ (5748)

5 sepsis of unknown origin.mp. (63)

6 sepsis of unknown origin.m_titl. (5)

7 sepsis of unknown cause.mp. (10)

8 sepsis of unknown cause.m_titl. (4)

9 sepsis of unknown source.mp. (1)

10 sepsis of unknown source.m_titl. (0)

11 5 or 6 or 7 or 8 or 9 or 10 (72)

12 2 or 3 or 4 (6185)

13 1 and 12 (329)

14 11 or 13 (396)

15 Diagnosis/ or diagnosis.mp. (3567487)

16 investigation.mp. (488658)

17 test.mp. (1487583)

18 examination.mp. or Physical Examination/ (687532)

19 History/ or history.mp. (954916)

20 approach.mp. (1194204)

21 imaging.mp. (2003537)

22 15 or 16 or 17 or 18 or 19 or 20 or 21 (8069724)

23 14 and 22 (268)

24 critically ill.mp. or Critical Illness/ (55196)

25 Intensive Care Units/ or ICU.mp. or Critical Care/ (127989)

26 24 or 25 (158400)

27 23 and 26 (29)

***************************

**Database: Pubmed 1951 to August 19, 2021**

Search Strategy:

((Sepsis) AND ((unknown source) OR (fever of unknown origin))) OR ((sepsis of unknown origin) OR (sepsis of unknown cause)) AND ((Diagnosis) OR (investigation) OR (test) OR (examination) OR (history) OR (approach) OR (imaging)) AND (critically ill)

(((sepsis of unknown origin) or (sepsis of unknown cause) or (sepsis of unknown source)) or ((sepsis) and ((unknown source) or (fever of unknown origin)))) and ((Diagnosis) or (investigation) or (test) or (examination) or (history) or (approach) or (imaging)) and ((critically ill patient) or (critically ill) or (intensive care))
