# Supplementary material for: Scoping review on diagnostic criteria and investigative approach in sepsis of unknown origin in critically ill patients
Source: J Intensive Care. 2022 Sep 11;10:44. doi: 10.1186/s40560-022-00633-4 (PMC9465866; doi:10.1186/s40560-022-00633-4)
Supplement: Supplementary file 2 — Additional file 2. Summary of Included Studies. Background information and extracted data from included studies. [file 40560_2022_633_MOESM2_ESM.docx]

**Summary of Included Studies**

| **Author(s)** | **Title** | **Type of Study** | **Year** | **Language** | **Country of origin** | **Definition of sepsis of unknown source** | **History** | **Examination** | **Imaging** | **Microbiological** | **Special** |
| --- | --- | --- | --- | --- | --- | --- | --- | --- | --- | --- | --- |
| Adam and Page [63] | Intra-abdominal sepsis: the role of radiology | Review | 1991 | English | United Kingdom | 0 | 0 | 0 | 1 | 1 | 0 |
| Agarwal *et al.* [23] | Epidemiology, risk factors and outcome of nosocomial infections in a Respiratory Intensive Care Unit in North India | Prospective cohort study | 2006 | English | India | 1 | 0 | 0 | 1 | 1 | 0 |
| Alberti *et al.* [17] | Influence of Systemic Inflammatory Response Syndrome and Sepsis on Outcome of Critically Ill Infected Patients | Prospective cohort study | 2003 | English | France | 0 | 0 | 0 | 0 | 0 | 0 |
| Baek *et al.* [88] | Four-Year Experience With Extracorporeal Membrane Oxygenation for Kidney Transplant Patients With Severe Refractory Cardiopulmonary Insufficiency | Retrospective cohort study | 2016 | English | Korea | 0 | 0 | 0 | 0 | 0 | 0 |
| Bajaj *et al.* [74] | High-grade fever and pancytopenia in an adult patient with common variable immune deficiency | Case report | 2014 | English | United States | 0 | 1 | 1 | 1 | 1 | 1 |
| Barkhausen *et al.* [26] | Impact of CT in patients with sepsis of unknown origin | Prospective cohort study | 1999 | English | Germany | 0 | 0 | 0 | 1 | 1 | 0 |
| Birndt *et al.* [24] | Cytokine adsorption in a patient with severe coagulation abnormalities due to hemophagocytic lymphohistiocytosis (HLH) | Case report | 2018 | English | Germany | 0 | 0 | 0 | 0 | 0 | 1 |
| Cengiz *et al.* [57] | Maxillary sinusitis in patients ventilated for a severe head injury and with nostrils free of any foreign body | Prospective cohort study | 2009 | English | Turkey | 0 | 0 | 0 | 1 | 1 | 0 |
| Cheung *et al.* [89] | A case of meropenem-induced liver injury and jaundice | Case report | 2021 | English | United States | 0 | 1 | 1 | 1 | 1 | 1 |
| Chueng *et al.* [37] | Case Report: Early Doxycycline Therapy for Potential Rickettsiosis in Critically Ill Patients in Flea-Borne Typhus-Endemic Areas | Case series | 2019 | English | United States | 0 | 1 | 1 | 1 | 1 | 1 |
| Contou *et al.* [16] | Septic shock with no diagnosis at 24 hours: a pragmatic multicenter prospective cohort study | Prospective cohort study | 2016 | English | France | 1 | 0 | 0 | 1 | 1 | 0 |
| Czupryna *et al.* [47] | Patients with sepsis in infectious diseases department in years 1997-2010 - epidemiology and clinical features. | Retrospective cohort study | 2013 | English | Poland | 0 | 1 | 1 | 0 | 0 | 0 |
| Dangoisse and Laterre [90] | Tracking the foreign body, a rare cause of hepatic abscess. | Case report | 2014 | English | Belgium | 0 | 0 | 1 | 1 | 1 | 0 |
| DeGiorgio *et al.* [91] | Atypical EBV encephalitis and extensive hemophagocytic lymphohistiocytic infiltrate in nervous system: A case report | Case report | 2016 | English | United States | 0 | 0 | 0 | 1 | 0 | 1 |
| Fabian *et al.* [92] | Septic shock due to self-administered stool suspensions in a patient with Munchausen syndrome. | Case report | 2018 | German | Germany | 0 | 0 | 0 | 1 | 0 | 0 |
| Fort *et al.* [25] | PET-CT in Critically Ill Patients: Diagnosing the Unsuspected | Case series | 2018 | English | France | 1 | 0 | 0 | 1 | 1 | 1 |
| Ghimire *et al.* [93] | Scenario of Sepsis in Intensive Care Unit in a Tertiary Care Hospital. | Prospective cohort study | 2020 | English | Nepal | 0 | 0 | 0 | 0 | 0 | 0 |
| Grundmann *et al.* [77] | The Acute (Surgical) Abdomen - Epidemiology, Diagnosis and General Principles of Management | Case report | 2010 | German | Germany | 0 | 0 | 0 | 1 | 0 | 1 |
| Haghighat *et al.* [94] | Fever of unknown origin post-cardiac transplantation | Case report | 2019 | English | United States | 0 | 0 | 0 | 0 | 0 | 1 |
| Haqiqi A. [51] | Purpura fulminans as a medical emergency, in a patient with angioimmunoblastic lymphoma | Case report | 2020 | English | United Kingdom | 0 | 0 | 1 | 1 | 0 | 1 |
| Heidelberg *et al.* [49] | An atypical case of necrotizing fasciitis secondary to perforated cecal cancer | Case report | 2020 | English | United States | 0 | 1 | 1 | 1 | 0 | 0 |
| Hindi *et al.* [52] | Hemophagocytic syndrome masquerading as septic shock: An approach to such dilemma | Case report | 2017 | English | United States | 0 | 0 | 0 | 1 | 1 | 1 |
| Hofmann *et al.* [71] | An unusual clinical course of Wegener's granulomatosis | Case report | 1998 | English | Austria | 0 | 0 | 0 | 1 | 1 | 1 |
| Huang *et al.* [64] | Diagnostic performance of FDG PET/CT in critically ill patients with suspected infection: A systematic review and meta-analysis | Systematic review | 2019 | English | Taiwan | 0 | 0 | 0 | 1 | 0 | 0 |
| Huang *et al.* [95] | Bilateral orbital compartment syndrome in a patient with disseminated intravascular coagulation | Case report | 2018 | English | Australia | 0 | 0 | 0 | 1 | 0 | 0 |
| Jafar N. [46] | Similar but not the same | Case report | 2019 | English | United States | 0 | 1 | 1 | 0 | 1 | 1 |
| Jane and Johnson [40] | Critical care nurses be aware: Lemierre's syndrome is on the rise | Review | 2003 | English | Australia | 0 | 1 | 1 | 1 | 1 | 0 |
| Jenkyn *et al.* [96] | Tip sparing tongue necrosis: A case report | Case report | 2017 | English | United Kingdom | 0 | 0 | 0 | 0 | 0 | 0 |
| Kampe *et al.* [65] | Diagnostic Value of Positron Emission Tomography Combined with Computed Tomography for Evaluating Critically Ill Neurological Patients | Retrospective cohort study | 2017 | English | Germany | 0 | 0 | 0 | 1 | 0 | 0 |
| Karanikolas *et al.* [72] | hyroid storm presenting as intra-abdominal sepsis with multi-organ failure requiring intensive care | Case report | 2009 | English | Greece | 0 | 0 | 1 | 1 | 0 | 1 |
| Kelly *et al.* [27] | The feasibility and accuracy of diagnostic laparoscopy in the septic ICU patient | Prospective cohort study | 2000 | English | United States | 1 | 0 | 0 | 0 | 0 | 1 |
| Klouwenberg *et al.* [18] | Likelihood of infection in patients with presumed sepsis at the time of intensive care unit admission: a cohort study | Prospective cohort study | 2015 | English | Netherlands | 0 | 0 | 0 | 0 | 0 | 0 |
| Kluge *et al.* [32] | Diagnostic value of positron emission tomography combined with computed tomography for evaluating patients with septic shock of unknown origin | Retrospective cohort study | 2012 | English | Germany | 1 | 0 | 0 | 1 | 1 | 0 |
| Kozlova *et al.* [76] | Multiple organ dysfunction syndrome in setting of sweet's syndrome without evidence of underlying infectious, rheumatologic, or oncologic etiology | Case report | 2019 | English | United States | 0 | 0 | 0 | 1 | 0 | 1 |
| Lee *et al.* [33] | Treatment of critically ill patients with sepsis of unknown cause: value of percutaneous cholecystostomy | Prospective cohort study | 1991 | English | United States | 1 | 0 | 0 | 1 | 1 | 1 |
| Legrand *et al.* [97] | Survival in neutropenic patients with severe sepsis or septic shock | Retrospective cohort study | 2012 | English | France | 0 | 0 | 0 | 0 | 0 | 0 |
| Lin *et al.* [42] | Secondary hemophagocytic lymphohistiocytosis in AIDS associated EBV+ diffuse large B-cell lymphoma | Case report | 2018 | English | United States | 0 | 1 | 0 | 1 | 1 | 1 |
| Lobo *et al.* [98] | Does the time of onset of severe sepsis in a surgical intensive care unit influence mortality rates: a single-center retrospective analysis | Prospective cohort study | 2010 | English | Germany | 0 | 0 | 0 | 0 | 0 | 0 |
| Maheshwari *et al.* [53] | Sepsis of unknown origin with multiorgan failure syndrome: Think of hemophagocytic lymphohistiocytosis. | Case report | 2015 | English | India | 0 | 1 | 1 | 0 | 1 | 1 |
| Mandry *et al.* [28] | 18F-fluorodeoxyglucose positron emission tomography combined with whole-body computed tomographic angiography in critically ill patients with suspected severe sepsis with no definite diagnosis | Prospective cohort study | 2014 | English | France | 1 | 0 | 1 | 1 | 1 | 0 |
| Mascia *et al.* [62] | Hemophagocytic Lymphohistiocytosis in Renal Transplant Recipients: A 2-Case Report | Case report | 2020 | English | Italy | 0 | 0 | 0 | 1 | 1 | 1 |
| Sexe *et al.* [38] | Euglycemic diabetic ketoacidosis in a lung cancer patient using empagliflozin | Case report | 2020 | English | United States | 0 | 1 | 0 | 0 | 0 | 0 |
| Minoja [29] | The clinical use of 99m-Tc-labeled WBC scintigraphy in critically ill surgical and trauma patients with occult sepsis | Prospective cohort study | 1996 | English | Italy | 1 | 1 | 0 | 1 | 1 | 0 |
| Muckart *et al.* [66] | Positron emission tomography/computed tomography scanning for the diagnosis of occult sepsis in the critically injured | Case Series | 2016 | English | South Africa | 0 | 0 | 0 | 1 | 1 | 0 |
| Neel *et al.* [50] | Diagnostic and management of life-threatening Adult-Onset Still Disease: a French nationwide multicenter study and systematic literature review | Case Series | 2018 | English | France | 0 | 1 | 1 | 0 | 0 | 1 |
| Okoli *et al.* [99] | The terrible triad: Hyperosmolar hyperglycemic state, hypertriglyceridemia, and acute pancreatitis in previously healthy patient | Case report | 2016 | English | United States | 0 | 1 | 0 | 1 | 0 | 0 |
| Padhi *et al.* [41] | Hemophagocytic lymphohistiocytosis: critical reappraisal of a potentially under-recognized condition | Case series | 2013 | English | India | 0 | 1 | 1 | 0 | 1 | 1 |
| Panjwani and Davis [34] | Malaria with acute respiratory distress syndrome: Developing country complications seen in America | Case report | 2018 | English | United States | 0 | 1 | 0 | 1 | 0 | 1 |
| Kansagra *et al.* [36] | Prolonged hypothermia due to olanzapine in the setting of renal failure: a case report and review of the literature | Case report | 2013 | English | United States | 0 | 1 | 0 | 1 | 1 | 1 |
| Peñuelas *et al.* [48] | Limb intracompartmental sepsis in burn patients associated with occult infection | Retrospective cohort study | 2010 | English | Spain | 0 | 0 | 1 | 0 | 1 | 0 |
| Peris *et al.* [30] | Bedside diagnostic laparoscopy to diagnose intraabdominal pathology in the intensive care unit | Retrospective cohort study | 2009 | English | Italy | 0 | 0 | 0 | 1 | 0 | 1 |
| Praetorius *et al.* [73] | Imported Anopheles: in the luggage or from the airplane? A case of severe autochthonous malaria tropica near an airport | Case report | 1999 | German | Germany | 0 | 1 | 0 | 1 | 0 | 1 |
| Reske *et al.* [100] | Kasuistik interaktiv: Lanzarote-Urlauber mit Sepsis und Multiorganversagen: Ein langer Weg bis zur richtigen Diagnose | Case report | 2010 | German | Germany | 0 | 1 | 0 | 1 | 0 | 1 |
| Riga *et al.* [101] | Rhinosinusitis in the intensive care unit patients: A review of the possible underlying mechanisms and proposals for the investigation of their potential role in functional treatment interventions. | Review | 2010 | English | Greece | 0 | 1 | 0 | 0 | 0 | 0 |
| Saevels [54] *et al.* | EBV-associated hemophagocytic lymphohistiocytosis complicated by severe coagulation disorders and opportunistic infections: case report of a survivor | Case report | 2017 | English | Belgium | 0 | 1 | 1 | 0 | 0 | 1 |
| Saleh *et al.* [102] | Is it really sepsis of unknown origin: a case of HLH | Case report | 2019 | English | United States | 0 | 1 | 0 | 1 | 0 | 1 |
| Samek *et al.* [60] | Monocenter study on epidemiology, outcomes, and risk factors of infections in recipients of 166 allogeneic stem cell transplantations during 1 year | Prospective cohort study | 2020 | English | Germany | 0 | 0 | 0 | 1 | 1 | 0 |
| Scawn *et al.* [75] | A pilot randomised controlled trial in intensive care patients comparing 7 days' treatment with empirical antibiotics with 2 days' treatment for hospital-acquired infection of unknown origin | Randomized controlled trial | 2012 | English | United Kingdom | 0 | 0 | 0 | 0 | 0 | 0 |
| Lichtenstein [56] | Point-of-care ultrasound: Infection control in the intensive care unit | Review | 2007 | English | France | 0 | 0 | 0 | 1 | 0 | 0 |
| Sheu *et al.* [103] | The influence of infection sites on development and mortality of ARDS | Prospective cohort study | 2010 | English | United States | 0 | 0 | 0 | 0 | 0 | 0 |
| Smith and Bodai [104] | Empyema of the gallbladder--potential consequence of medical intensive care | Case series | 1982 | English | United States | 0 | 0 | 1 | 1 | 1 | 0 |
| Stelter *et al.* [58] | Computed tomography findings in septic patients with acute respiratory distress syndrome: correlation with survival and pulmonary versus extrapulmonary septic focus | Prospective cohort study | 2013 | English | Germany | 0 | 0 | 0 | 1 | 0 | 0 |
| Takano *et al.* [39] | Liver abscessation and multiple septic pulmonary emboli associated with Lemierre's syndrome: a case report | Case report | 2015 | English | Japan | 0 | 1 | 1 | 1 | 1 | 0 |
| Tanguy M [68] | Hub qualitative blood culture is useful for diagnosis of catheter-related infections in critically ill patients | Prospective cohort study | 2005 | English | France | 0 | 0 | 0 | 0 | 1 | 0 |
| Tonolini and Ippolito [59] | Cross-sectional imaging of complicated urinary infections affecting the lower tract and male genital organs | Review | 2016 | English | Italy | 0 | 0 | 0 | 1 | 0 | 0 |
| Urli *et al.* [69] | Surveillance of infections acquired in intensive care: usefulness in clinical practice | Prospective cohort study | 2002 | English | Italy | 0 | 0 | 0 | 0 | 1 | 0 |
| van de Hoven *et al.* [35] | Azathioprine hypersensitivity syndrome, a drug reaction mimicking sepsis | Case report | 2020 | English | Netherlands | 0 | 1 | 1 | 1 | 1 | 1 |
| Hulst *et al.* [105] | The value of F-18-fluorodeoxyglucose positron emission tomography (FDG-PET/CT) in the intensive care unit: A review | Review | 2019 | English | Netherlands | 1 | 0 | 0 | 1 | 1 | 0 |
| Velmahos *et al.* [31] | Abdominal computed tomography for the diagnosis of intra-abdominal sepsis in critically injured patients: fishing in murky waters | Case series | 1999 | English | United States | 1 | 0 | 0 | 1 | 0 | 0 |
| Seiden [61] | Sinusitis in the critical care patient | Review | 1993 | English | United States | 0 | 1 | 0 | 1 | 1 | 0 |
| Green *et al.* [106] | Investigating the causes of fever in critically ill patients. Are you overlooking noninfectious causes? | Review | 1995 | English | United States | 0 | 0 | 0 | 0 | 0 | 0 |
| Kluge *et al.* [107] | Pylephlebitis with air in the portal vein system. An unusual focus in a patient with sepsis | Case report | 2003 | English | Germany | 0 | 1 | 1 | 1 | 0 | 0 |
| Darwich and Gleeson [67] | Fever of unknown origin in the ICU: Key components of the work-up | Review | 1998 | English | United States | 0 | 1 | 0 | 1 | 1 | 0 |
| Drewek-Platena *et al.* [108] | Sepsis and SIRS: A survey based on the data of the ICU and IMC-ward of Klinikum Lippe-Detmold in 2011 | Prospective cohort study | 2011 | English | Germany | 0 | 0 | 0 | 0 | 0 | 0 |
| Duguid and Koutsavlis [109] | Critical care of patients with haematological malignancies-a retrospective analysis of one tertiary centre's experience | Retrospective cohort study | 2016 | English | United Kingdom | 0 | 0 | 0 | 0 | 0 | 0 |
| Edmondson*et al.* [110] | Patients with haematological malignancy requiring intensive care treatment: A 5 year review | Retrospective cohort study | 2012 | English | United Kingdom | 0 | 0 | 0 | 0 | 0 | 0 |
| Holmes [111] | Bacteroides fragilis-an unlikely pathogen infecting a hip prosthesis | Case report | 2019 | English | United Kingdom | 0 | 1 | 0 | 1 | 0 | 0 |
| Ivory *et al.* [70] | Hide and go seek: A case of vancomycin-resistant enterococcus in the bone marrow | Case report | 2019 | English | United States | 0 | 0 | 0 | 0 | 1 | 0 |
| Kapoor *et al.* [44] | A rare case of EBV induced hemophagocytic lymphohistocytosis | Case report | 2014 | English | United States | 0 | 1 | 1 | 1 | 1 | 1 |
| Khaliq *et al.* [45] | Walks like sepsis, talks like sepsis, but still not sepsis: Hematophagocytic lymphohistiocytosis | Case report | 2012 | English | United States | 0 | 1 | 1 | 1 | 1 | 1 |
| Klutmann *et al.* [112] | The role of PET/CT for diagnosis of sepsis in the intensive care unit | Retrospective cohort study | 2010 | English | Germany | 0 | 0 | 0 | 1 | 0 | 0 |
| McDowell and Dawson [113] | Evaluation of the abdomen in sepsis of unknown origin | Review | 1996 | English | United States | 0 | 0 | 0 | 1 | 0 | 0 |
| McFarlane *et al.* [114] | Hospitalized influenza patients during 2013-2014: A comparison of ICU and ward treated patients including antimicrobial therapy, adverse events, and outcomes | Retrospective cohort study | 2015 | English | Canada | 0 | 0 | 0 | 0 | 0 | 0 |
| Rodrigues [78] | Cholecystostomy | Review | 2009 | English | Portugal | 0 | 0 | 0 | 0 | 1 | 0 |
| Ngo and Nguyen [43] | Certainly sirs, but sepsis misses the sweet spot | Case report | 2015 | English | United States | 0 | 1 | 1 | 1 | 1 | 1 |
| Panknin and Kindgen-Milles [115] | Fever of unknown origin on intensive care units: Common causes and diagnostic procedures | Review | 2000 | English | Germany | 0 | 0 | 0 | 0 | 0 | 0 |
| Theerawit *et al.* [116] | Hemodynamic disclosure of septic shock patients by intensive care ultrasound | Prospective cohort study | 2013 | English | Thailand | 0 | 0 | 0 | 0 | 0 | 0 |
| Ulibarrena *et al.* [117] | Scintigraphy with labeled leucocytes in the diagnosis of an infectious source in the critical patient | Case series | 1997 | English | Spain | 0 | 0 | 0 | 1 | 0 | 0 |
| Balík [55] | Importance of ultrasound examination in diagnosing acute conditions | Case report | 2019 | English | Czech Republic | 0 | 0 | 0 | 1 | 0 | 0 |
