# Supplementary material for: Scoping review on diagnostic criteria and investigative approach in sepsis of unknown origin in critically ill patients
Source: J Intensive Care. 2022 Sep 11;10:44. doi: 10.1186/s40560-022-00633-4 (PMC9465866; doi:10.1186/s40560-022-00633-4)
Supplement: Supplementary file 3 — Additional file 3. Diagnostic Workup Extraction. Extraction of data on diagnostic thematic processes from articles. [file 40560_2022_633_MOESM3_ESM.docx]

**Diagnostic Workup Extraction**

This list contains items under each diagnostic thematic process that were used during extraction of data from included articles. To qualify for a thematic process (history, examination, imaging, microbiology, special tests), an article needs to include at least one of the items under the thematic process and the item must be described as part of the diagnostic workup of patients with suspected sepsis of unknown origin.

| **History** |
| --- |
| Respiratory (cough, sputum, dyspnea, chest pain, hemoptysis)  Gastrointestinal (pain, vomiting, diarrhea, jaundice, distension, stool)  Urinary (incontinence, hematuria, dysuria, cloudy urine)  Neurological (headache, neck pain, confusion, seizures, hallucinations)  Musculoskeletal (arthralgia, joint swelling/erythema, neck/back pain)  Ear Nose Throat (dysphagia, odynophagia, oral ulcers, epistaxis, trismus, toothache)  Systemic (weight loss, night sweats, fever, malaise, rigors)  Immunosuppression (HIV, chemotherapy, diabetes mellitus, cancer, drugs)  Past medical history  Family history  Drug history  Collateral history  Urology/Gynecological/Obstetrics (sexual history, pregnancy, tampon use, pelvic pain)  Pets/Animal Contact  Occupation  Travel/Contact |

| **Examination** |
| --- |
| Respiratory  Cardiovascular  Abdominal  Neurological  Lymph Nodes (all regions)  Musculoskeletal  Perineum  Nail  Skin  Oral, nasal, ears, sinus, neck  Catheter Sites  Temperature |

| **Imaging** |
| --- |
| Echocardiography  Ultrasonography  Computer Tomography  X-ray/Plain radiograph  Magnetic Resonance Imaging  Radioisotope White Cell Scan  Positron Emission Tomography Computer Tomography |

| **Microbiology** |
| --- |
| Any Body Fluid Culture (blood, urine, sputum, tracheal aspirate, cerebral spinal fluid, joint, bone marrow, pleural, ascitic, abscess)  Prosthesis/Catheter tip culture  Bronchoalveloar lavage  Stool culture |

| **Special Tests** |
| --- |
| Amylase  Immunoglobulin Level  Ferritin  B12 Folate  Serum Triglyceride  Fibrinogen  Lactate Dehydrogenase  C-reactive Protein  Erythrocyte sedimentation rate  Procalcitonin  Autoimmune antibodies  Cortisol Level  Thyroid hormones  Drug Toxicology  Bone Marrow Examination  Biopsy of lymph node  Peripheral blood smear (for pathogens such as malaria, leishmaniases and hemolysis)  Flow cytometry  Diagnostic laparoscopy  Skin biopsy  Percutaneous cholecystostomy  Pathogen specific antigens (galactomannan, cryptococcal, streptoccoal, legionella)  Viral DNA (CMV, Hepatitis B, Hepatitis C, EBV)  Pathogen Antibody/Serology (Dengue, Syphilis, Monospot, Weil-Felix test, viral hepatitis, HIV)  QuantiFERON for *Myocabacterium tuberculosis*  Virus Polymerase Chain Reaction |

CMV, Cytomegalovirus; EBV, Epstein-Barr virus; DNA, Deoxyribonucleic acid; HIV, Human immunodeficiency virus;
